# Terminally exhausted CD8^+^ T cells contribute to age-dependent severity of respiratory virus infection

**DOI:** 10.1186/s12979-023-00365-5

**Published:** 2023-08-01

**Authors:** Olivia B. Parks, Taylor Eddens, Jorna Sojati, Jie Lan, Yu Zhang, Tim D. Oury, Manda Ramsey, John J. Erickson, Craig A. Byersdorfer, John V. Williams

**Affiliations:** 1 Department of Pediatrics, Division of Infectious Diseases, University of Pittsburgh School of Medicine, Pittsburgh, PA USA; 2 Department of Pediatrics, Division of Allergy/Immunology, University of Pittsburgh School of Medicine, Pittsburgh, PA USA; 3 Department of Pathology, University of Pittsburgh School of Medicine, Pittsburgh, PA USA; 4 Department of Pediatrics, Division of Blood and Marrow Transplant and Cellular Therapies, University of Pittsburgh School of Medicine, Pittsburgh, PA USA; 5 Department of Pediatrics, Division of Neonatology and Pulmonary Biology, Cincinnati Children’s Hospital Medical Center, University of Cincinnati School of Medicine, Cincinnati, OH USA; 6 Institute for Infection, Inflammation, and Immunity in Children (i4Kids), Pittsburgh, PA USA; 7 University of Pittsburgh, Rangos Research Building, 4401 Penn Avenue, Pittsburgh, PA 15224 USA

**Keywords:** Respiratory viral infection, Aged immune response, Viral immunology

## Abstract

**Background:**

Lower respiratory infections are a leading cause of severe morbidity and mortality among older adults. Despite ubiquitous exposure to common respiratory pathogens throughout life and near universal seropositivity, antibodies fail to effectively protect the elderly. Therefore, we hypothesized that severe respiratory illness in the elderly is due to deficient CD8^+^ T cell responses.

**Results:**

Here, we establish an aged mouse model of human metapneumovirus infection (HMPV) wherein aged C57BL/6 mice exhibit worsened weight loss, clinical disease, lung pathology and delayed viral clearance compared to young adult mice. Aged mice generate fewer lung-infiltrating HMPV epitope-specific CD8^+^ T cells. Those that do expand demonstrate higher expression of PD-1 and other inhibitory receptors and are functionally impaired. Transplant of aged T cells into young mice and vice versa, as well as adoptive transfer of young versus aged CD8^+^ T cells into *Rag1*^*−/−*^ recipients, recapitulates the HMPV aged phenotype, suggesting a cell-intrinsic age-associated defect. HMPV-specific aged CD8^+^ T cells exhibit a terminally exhausted TCF1/7^−^ TOX^+^ EOMES^+^ phenotype. We confirmed similar terminal exhaustion of aged CD8^+^ T cells during influenza viral infection.

**Conclusions:**

This study identifies terminal CD8^+^ T cell exhaustion as a mechanism of severe disease from respiratory viral infections in the elderly.

**Supplementary Information:**

The online version contains supplementary material available at 10.1186/s12979-023-00365-5.

## Background

Respiratory viral infections are a leading cause of mortality worldwide, occurring predominantly in children < 2 years, adults > 65 years, and the immunocompromised [1]. Human metapneumovirus (HMPV) is a leading cause of acute lower respiratory infections, with nearly 100% of children becoming seropositive by age 5 [1]. Despite universal early exposure, re-infections of HMPV occur throughout adult life, but usually result in mild, self-resolving respiratory disease [1]. However, elderly individuals infected with HMPV are at risk for increased morbidity and mortality associated with HMPV pneumonia and both bacterial and viral co-infections; in some studies, HMPV is as common among older adults as influenza and respiratory syncytial virus (RSV) [2–5].

There are limited data on HMPV immune responses and pathogenesis in humans, primarily in young children [6–9]. Previous studies using young adult mouse HMPV models showed that CD8^+^ T cells help mediate viral clearance and protective immunity [10–13]. However, CD8^+^ T cell responses are constrained during acute respiratory virus infection by coordinated signaling of cell surface inhibitory receptors, predominantly Programmed cell death-1 (PD-1) and lymphocyte activation gene 3 (LAG-3), which remain upregulated even following viral clearance [10]. The persistent co-expression of these receptors, particularly PD-1, leads to a state of functional impairment during acute respiratory virus infection manifested by diminished degranulation and cytokine production such as IFNγ, and delayed viral clearance [10–13].

The aging immune system is characterized by a functional decline in virtually all immune cells, coupled with an increase in basal pro-inflammatory cytokine production, termed “inflammaging” [14, 15]. High throughput analysis at the single cell level has revealed distinct transcriptional and epigenetic changes in immune cells in both mice and humans as they age [16, 17]. Bulk CD8^+^ T cells in aged mice increase expression of the transcription factors thymocyte selection-associated high mobility group box protein (TOX) and eomesodermin (EOMES), changes that promote terminal differentiation of CD8^+^ T cells [16, 18, 19]. A subset of murine CD8^+^ T cells increase production of granzyme K with increased age, which contributes to the inflammatory microenvironment [16]. In addition, murine CD8^+^ tissue-resident memory T cells (T_RM_) have been identified as the cause of lung inflammation and fibrosis during viral pneumonia in aged mice [20]. CD8^+^ T cell responses to influenza have been studied in aged mice [21, 22] and older humans [23–25]. Low granzyme B production by influenza-specific cytolytic T lymphocytes (CTL) from elderly humans correlated with lower protection against influenza and has been considered a marker for optimal protective vaccine response in humans [23, 24]. Another report described decreased degranulation but lower PD-1 expression on influenza-specific CD8^+^ T cells from aged humans [24, 25]. However, one limitation of human studies is the use of viral peptide stimulation instead of tetramers; which, while widely used, fails to detect unresponsive virus-specific CD8^+^ T cells.

We developed an aged mouse model for HMPV to explore the contribution of both age and virus-specific CD8^+^ T cells to protection and disease. We found that aged HMPV-infected mice exhibited worse clinical disease, delayed viral clearance, and enhanced lung pathology compared with young mice. Virus-specific CD8^+^ T cells in aged mice were fewer, expressed higher levels of PD-1 and other inhibitory receptors, and exhibited markedly decreased granzyme B production. Reciprocal transplant of aged T cells into young mice and vice versa, as well as adoptive transfer of aged or young CD8^+^ T cells into *Rag1*^*−/−*^ mice, confirmed this was a CD8^+^ T cell-intrinsic age-associated defect. Similarly, influenza-infected aged mice lost more weight, produced fewer tetramer^+^ CD8^+^ T cells, had impaired granzyme B production, and accumulated more TCF1/7^−^ TOX^+^ EOMES^+^ CD8^+^ T cells. Collectively, these results identify a population of dysregulated and dysfunctional terminally exhausted CD8^+^ T cells in the aged host which contributes to the severity of multiple respiratory viral diseases in this population.

## Results

### HMPV-infected aged mice exhibit more severe disease

**Fig. 1:**
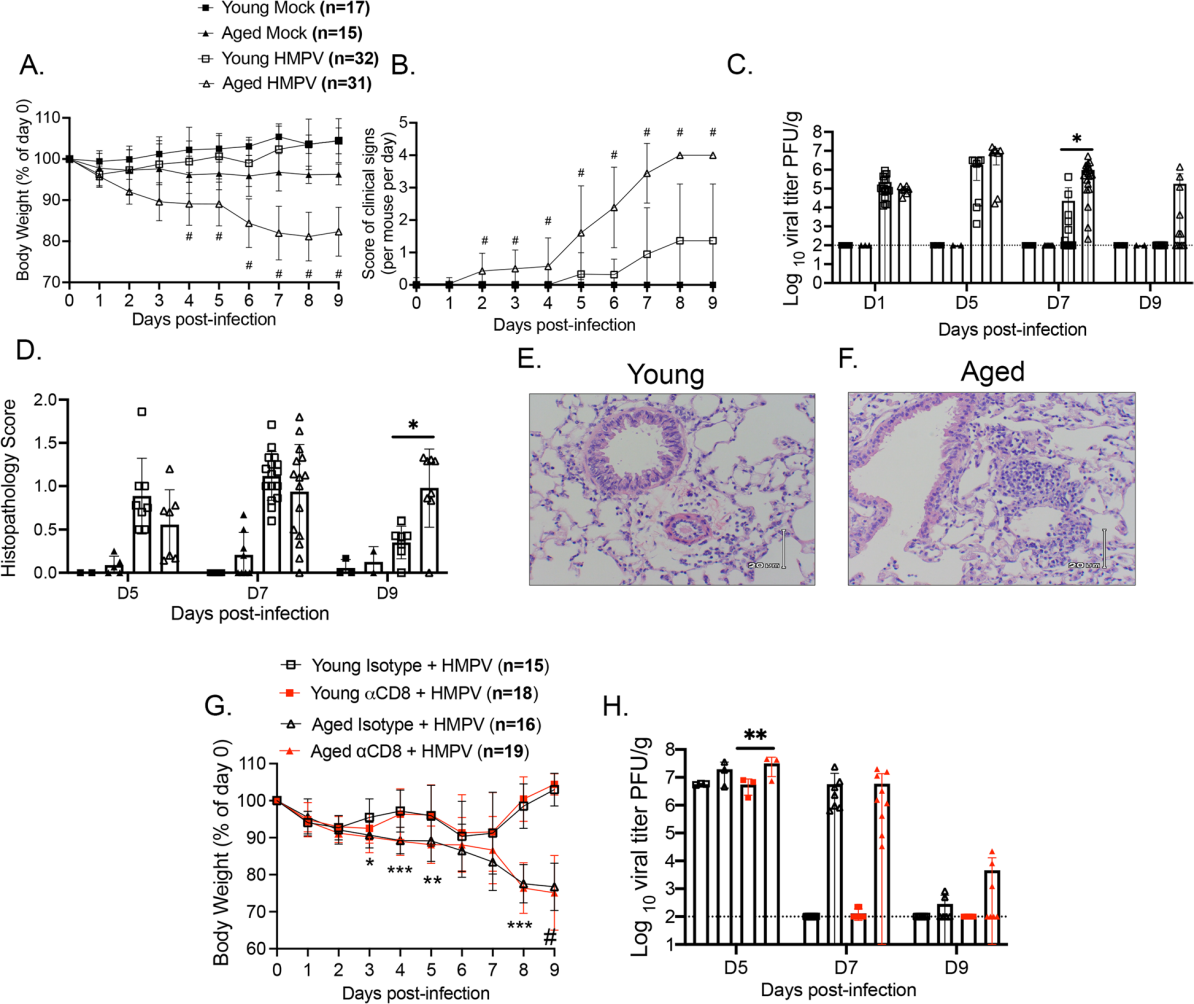
Aged mice had more severe HMPV disease and delayed viral clearance. **A** Aged infected mice lost more weight during HMPV infection, *P* < 0.0001 days 4–9 p.i. for aged vs. young HMPV. **B** Aged mice had more severe clinical signs throughout infection. Clinical signs included: hunched, rapid breathing, lethargy, and ruffled fur with one point given for each sign observed. Two individuals independently scored each mouse daily and average score was calculated, *P* < 0.0001 on day 2–9 p.i. for aged vs. young HMPV. **C** Aged mice had delayed HMPV clearance. Viral titer in lung in PFU/g was quantified using plaque assay and normalized to lung weight. Dotted line indicates limit of detection (LOD). **D** Aged infected mice accumulated more inflammatory infiltrates in the perivascular and peribronchial spaces in the lung by day 9 p.i. The histopathology score: average score per section field by a group-blinded experienced lung pathologist. 0 = no inflammation, 1 =  < 25% inflammation, 2 = 25–50% inflammation, 3 = 50–75% inflammation, and 4 =  > 75% inflammation. **E** &** F** Representative lung histology at day 9 p.i. **G** Aged and young TN/94–49 HMPV infected mice were treated with either αCD8 blocking antibody (Ab) or rat isotype control Ab. Aged vs. young isotype groups weight loss significant at days 3, 4, 8, and 9 p.i; aged vs. young αCD8 groups significant at days 4, 5, 8, and 9 p.i. **H** Aged αCD8 mice tended to have delayed viral clearance compared to young αCD8 mice. Dotted line indicates LOD. Number of mice per group at D5, D7, and D9, respectively: Aged isotype (*n* = 3,7,6), Young isotype (*n* = 3,7,6), Aged αCD8 (*n* = 3, 9, 7), Young αCD8 (*n* = 3,8,7). Number of mice per group below LOD at D5, D7, and D9, respectively: Aged isotype (*n* = 0/3,0/7,4/6), Young isotype (*n* = 0/3,7/7,6/6), Aged αCD8 (*n* = 0/3,0/9,4/7), Young αCD8 (*n* = 0/3,6/8,7/7). *P < 0.05,**P < 0.01, ***P < 0.001, # = ****P < 0.0001, one or two-way ANOVA

To establish an aged mouse model for HMPV, young (6-7wks) and aged (70-71wks) C57BL/6 (B6) mice were infected with HMPV TN/94–49. This strain causes mild, self-resolving disease in young adult B6 mice with minimal weight loss, transient lung inflammation, and viral clearance by day 7–9 post-infection (p.i.) [10–13]. Young infected mice in the current experiments similarly exhibited a mild phenotype, while aged infected mice lost significantly more weight (Fig. 1A) and displayed higher clinical illness scores (Fig. 1B). Aged mice had higher viral titers at day 7 p.i. and delayed viral clearance, with 50% of aged mice having detectable viral titer at day 9 p.i. (Fig. 1C). Moreover, aged mice retained enhanced lung inflammation late in HMPV infection as evidenced by peribronchial and perivascular infiltrates and increased histopathology scores (Fig. 1D-F).

CD8^+^ T cells contribute to viral clearance of HMPV in young mice [10–13]. To test the role that CD8^+^ T cells play in aged mice, CD8^+^ T cells were depleted from young and aged mice. Mice received αCD8 depleting antibody (Ab) or isotype control Ab via intraperitoneal injection one day prior to HMPV infection and every other day p.i.. CD8^+^ depletion was confirmed via flow cytometry with representative flow plots shown at day 5 p.i. (Supp. Figure 1A). Aged mice again lost significantly more weight compared to young mice, but CD8^+^ depletion did not result in further weight loss in either age group (Fig. 1G). CD8 depletion in aged mice had no major impact on viral titer, although aged mice depleted of CD8^+^ T cells tended to have higher viral titers on days 5 p.i. compared to young CD8^+^ depleted mice and a trend towards higher titer on day 9 p.i. compared to aged isotype control (Fig. 1H). There was no difference in CD19^+^ B220^+^ B cells between isotype and CD8^+^ depleted groups of either age (Supp. Figure 1B & C). However, there was a significant increase in CD4^+^ T cell absolute number in aged CD8 depleted mice (Supp. Figure 1C). The lack of differences in young mice with CD8^+^ depletion is most likely because HMPV viral clearance is a multifactorial process. A prior study found that CD4^+^ T cells could compensate for a lack of CD8^+^ T cells and contribute to viral clearance in young mice infected with HMPV [26]. Collectively, these data reveal enhanced susceptibility and more severe clinical illness in aged HMPV-infected mice with a trend towards delayed viral clearance in aged mice depleted of CD8^+^ T cells.

### Aged mice generate fewer HMPV-specific CD8^+^ T cells in the lung

**Fig. 2:**
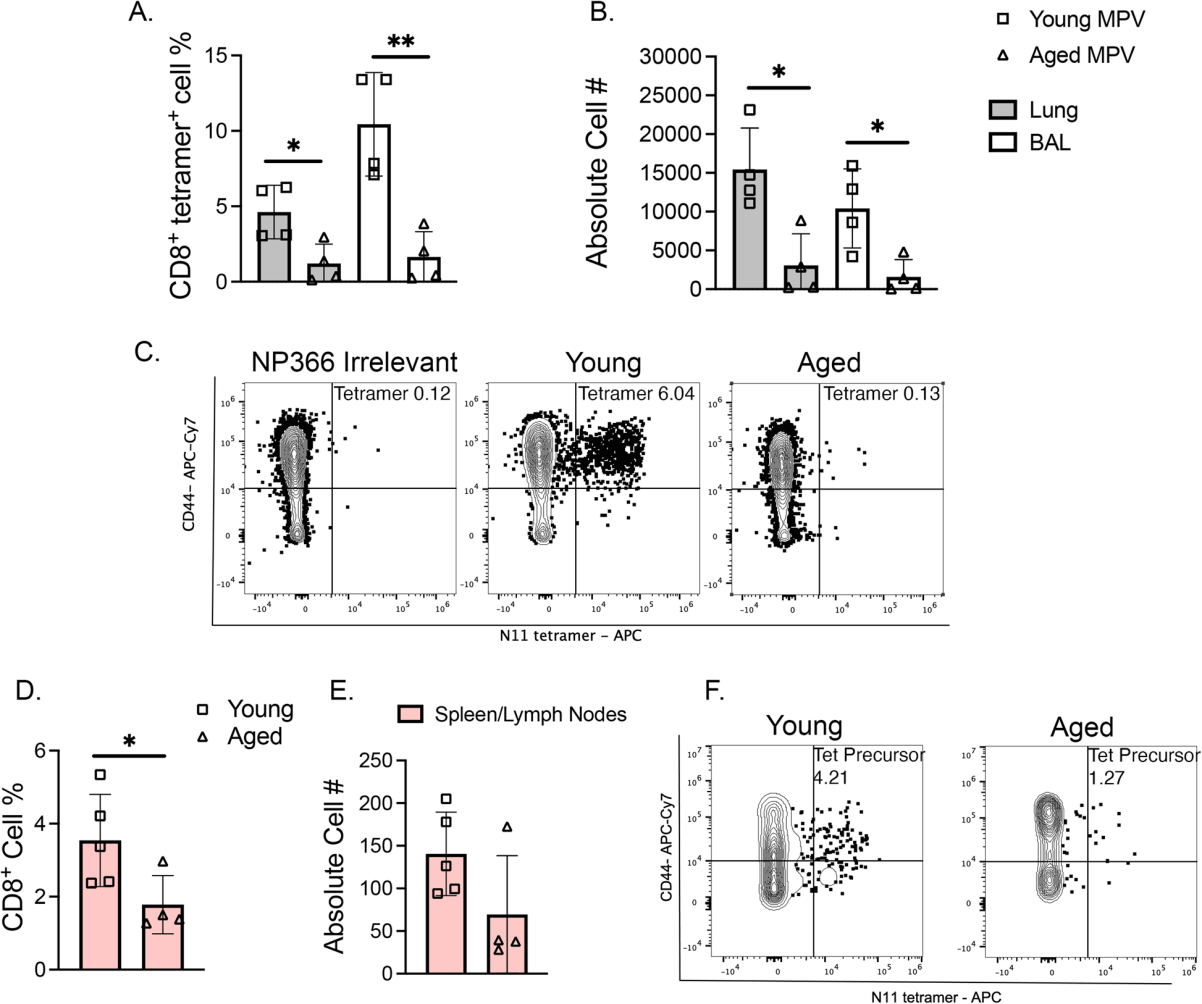
Aged mice produce fewer HMPV-specific CD8 + T cells in the lung. **A**, **B** Aged infected mice had decreased CD8^+^ N11 tetramer^+^ cells in lung (shaded bars) and BAL (open bars) compared to young infected mice at day 7 post-infection. Both N11 percent of CD8^+^ tetramer^+^ T cells and absolute cell number is shown. Absolute cell number was calculated by Biolegend Precision Counting Beads. **C** Representative flow plots from young and aged infected lung, respectively. **D**-**E** Aged uninfected mice had fewer tetramer precursor cells in the spleen and lymph nodes compared to young uninfected mice. Tetramer precursors were enriched from uninfected mouse spleen and lymph node via magnetic column selection using APC-labeled beads. **F** Representative flow plots of CD8^+^ tetramer precursor cells shown. *P < 0.05, **P < 0.01, unpaired t-test

We next quantified HMPV-specific CD8^+^ T cell responses in the lungs of young versus aged mice. We used MHC-I tetramers bearing one of the immunodominant H2-K^b^ HMPV epitopes, N_11-18_ [10]. At day 7 post-infection, aged HMPV infected mice produced fewer epitope-specific CD8^+^ tetramer^+^ (tet^+^) T cells compared to young infected mice in both lung and bronchoalveolar lavage (BAL) (Fig. 2A-C). Similar findings were observed for a different HMPV epitope, M_94-102_, with a significant decrease in tet^+^ percent in both lung and BAL, but only a trend in absolute cell number (Supp. Figure 2). These findings demonstrate that the reduced number of HMPV-specific cells in aged mice was not unique to a single epitope. While virus-specific CD8^+^ T cells were reduced in aged mice, the absolute cell number of bulk CD8^+^ T lymphocytes was not different between the two groups (Supp. Figure 3A). These findings suggest that changes in aged mice were specific to infection-induced lung-infiltrating, virus-reactive CD8^+^ T cells. Similarly, no difference in absolute cell number of CD8^+^ tet^+^ T cells was noted in the draining lymph nodes or spleen between young and aged mice, though there were modestly increased but rare tetramer-specific cells in the aged spleens (Supp. Figure 3B-D). These findings indicate that aged mice primed HMPV-specific CD8^+^ T cells to a similar degree in lymphoid organs compared to young HMPV-infected mice and did not exhibit migration defects.

To test whether aged mice possess reduced epitope-specific CD8^+^ T cell precursors or an intrinsic ability to differentiate into effector cells, we performed a tetramer enrichment of spleen and peripheral lymph nodes (combined to facilitate detection of rare precursors) in naïve aged and young mice. Aged mice had fewer epitope-specific precursor CD8^+^ T cells compared to young mice (Fig. 2D-F). We then quantified total CD44^−^ CD62L^+^ naïve CD8^+^ T cells (T_N_) in the lung of naïve aged and young mice and found that aged mice had substantially fewer T_N_ compared to young mice (Supp Fig. 4A-B). Upon infection, aged mice failed to robustly increase CD44^+^ CD62L^−^ T effector CD8^+^ T cells (T_EM_) (Supp Fig. 4A-C). Collectively, these data indicate significantly reduced naïve CD8^+^ T cell precursors and diminished capacity to differentiate into effector cells in aged mice.

### Aged HMPV-specific CD8^+^ T cells co-express multiple inhibitory receptors and produce significantly less granzyme B

**Fig. 3:**
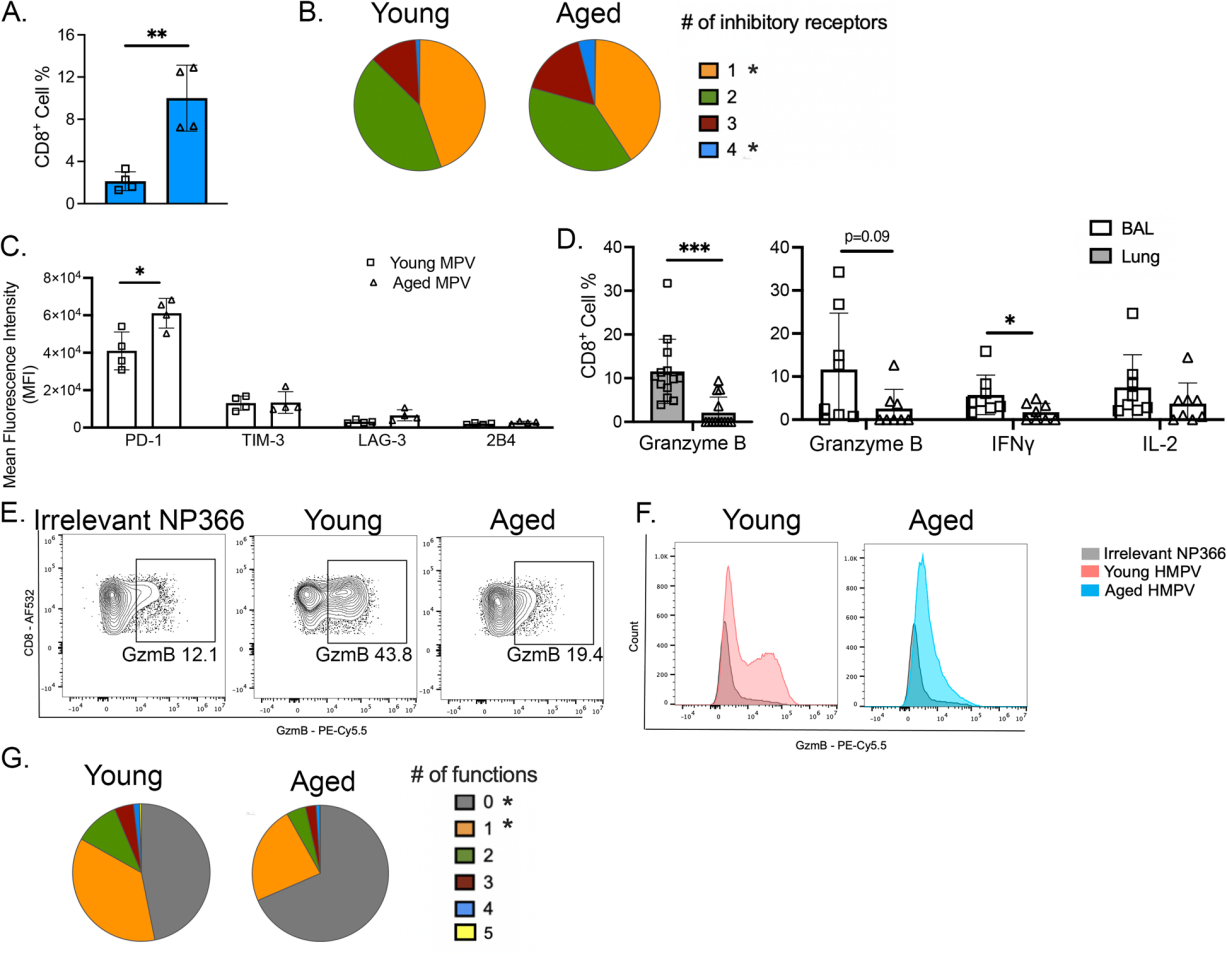
Aged HMPV-specific CD8^+^ T cells co-express inhibitory receptors and produce less granzyme B. **A** CD8^+^ N11 tet^+^ cells from aged infected mice had increased expression of all 4 inhibitory receptors PD-1, TIM-3, LAG-3, and 2B4 compared to young infected mice. **B** Pie charts represent SPICE software analysis of inhibitory receptor co-expression on CD8^+^ N11 tet^+^ T cells in BAL of infected aged and young mice on day 7 post-infection. Asterisks indicate statistically significant differences between aged and young. **C** Aged CD8^+^ tet^+^ cells in BAL had increased mean fluorescence intensity (MFI) of PD-1 at day 7 post-infection while there was no difference in MFI in other inhibitory receptors. **D** CD8^+^ cells from aged infected mice had decreased production of granzyme B in lung (shaded bars). In BAL (open bars) aged CD8^+^ T cells produced significantly less IFNγ with a trend towards decreased production of granzyme B and IL-2. **E** & **F** Representative flow plots and histograms of granzyme B production in young and aged infected lung at day 7 post-infection with NP366 flu irrelevant as control. **G** Aged CD8^+^ T cells in lung were less polyfunctional. Functional markers measured: granzyme B, IFNγ, TNF, perforin, and CD107a. *P < 0.05, **P < 0.001, unpaired t-test

In addition to quantitative differences and changes in differentiation potential of virus-specific CD8^+^ T cells, we sought to characterize functional attributes of aged compared with young CD8^+^ T cells. We previously showed in young adult mice that lung CD8^+^ tet^+^ cells upregulated numerous inhibitory receptors by day 7 p.i. and that PD-1 significantly contributed to CD8^+^ T cell functional impairment [10–13]. The fraction of HMPV N11-specific CD8^+^ T cells co-expressing all four inhibitory receptors PD-1, TIM-3, LAG-3, and 2B4 increased in the BAL of aged mice at day 7 post-infection (Fig. 3A & B blue pie slice). The number of CD8^+^ tet^+^ T cells expressing just one inhibitory receptor (orange pie slice) was also increased in aged CD8^+^ tet^+^ T cells (Fig. 3B). Additionally, aged HMPV-specific CD8^+^ cells expressed more PD-1 on a per cell basis as measured by mean fluorescence intensity (MFI) (Fig. 3C). These results indicate that aged virus-specific CD8^+^ T cells more highly co-express the inhibitory receptors PD-1, TIM-3, LAG-3, and 2B4, which could contribute to decreased ability of these cells to combat infection [10–13].

To test whether aged CD8^+^ T cells were functionally impaired during HMPV infection, we quantified intracellular cytokine production and degranulation in aged CD8^+^ T cells from infected mice following restimulation with HMPV epitope peptides [10]. Following restimulation, aged CD8^+^ T cells displayed significantly decreased granzyme B production compared to young CD8^+^ T cells in the lung, with a similar trend in BAL (Fig. 3D). There was also decreased production of IFNγ by aged CD8^+^ T cells in BAL (Fig. 3D). Representative flow plots and histograms of granzyme B production in the lung are shown in Fig. 3E & F. Combinatorial analysis based on expression of granzyme B, IFNγ, TNF, CD107a, and perforin revealed that aged CD8^+^ T cells were significantly less polyfunctional with many more aged cells exhibiting zero measured functions (Fig. 3G). Taken together, these data indicate not only are HMPV-specific CD8^+^ T cells quantitatively reduced in aged mice, but they also co-express numerous inhibitory receptors and are functionally impaired.

### Aged T cells transplanted into young mice recapitulate the aged immune response to HMPV

**Fig. 4:**
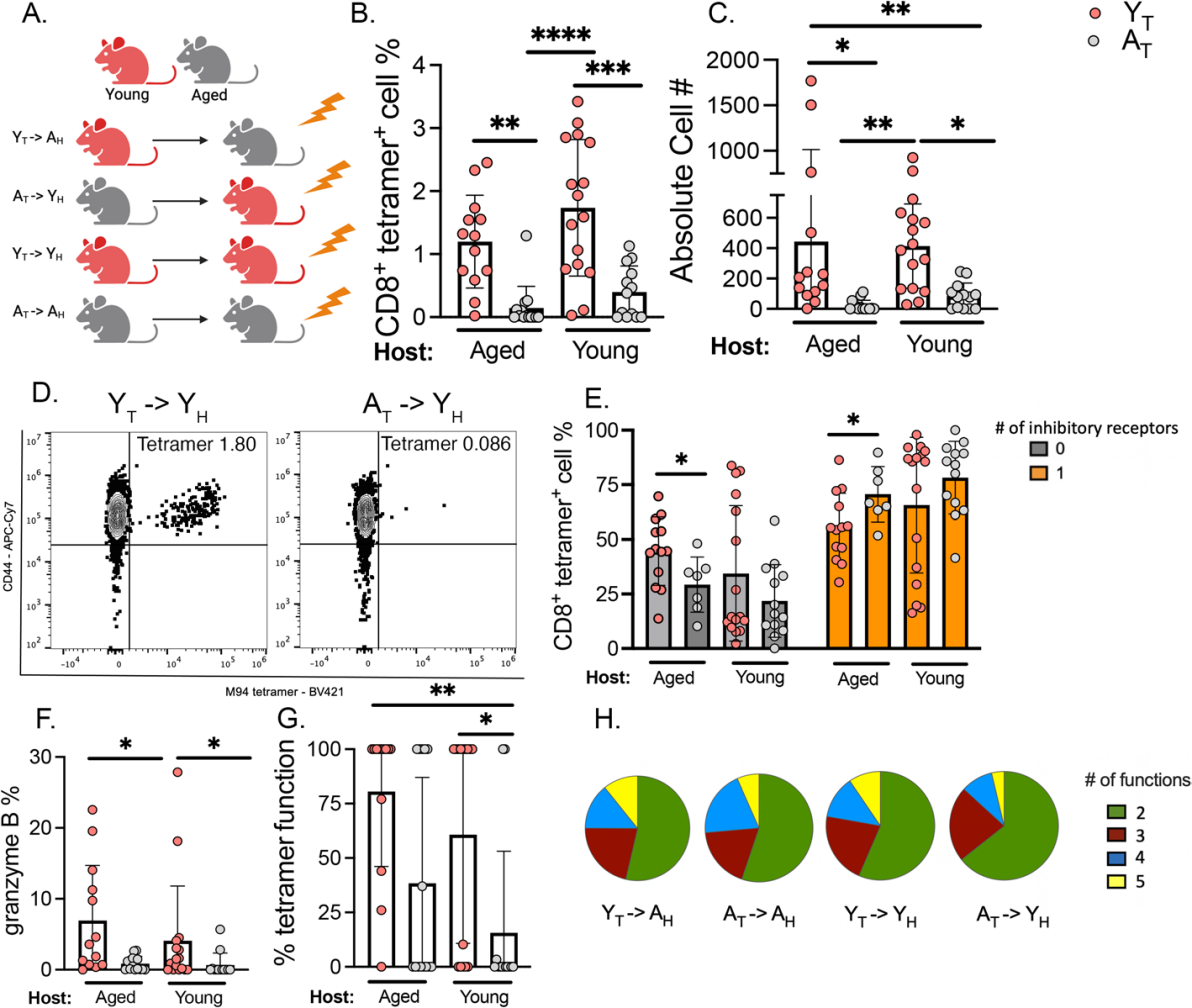
Aged T cells transplanted into young mice recapitulated aged CD8^+^ T cell phenotype. **A** Syngeneic transplant experimental design. Lethally irradiated CD45.2 aged mice were reconstituted with T lymphocytes from young or aged CD45.1 donors, young B cells, and *Rag1*^*−/−*^ BM. **B**, **C** Aged mice that received young T lymphocytes (i.e. Y_T_—> A_H_) had improved CD8^+^ tetramer^+^ response compared to A_T_—> A_H_ control. A_T_—> Y_H_ had a diminished tetramer response compared to Y_T_—> Y_H_ **D** Representative flow plots of tetramer staining. **E** Combinatorial analysis of PD-1, TIM-3, LAG-3, and 2B4 inhibitory receptors revealed that Y_T_—> A_H_ had increased percent of CD8^+^ T cells that expressed 0 inhibitory receptors and decreased percent of CD8^+^ T cells that expressed at least 1 inhibitory receptor compared to A_T_—> A_H_. **F** Percentage of CD8^+^ T cells expressing granzyme B. **G** Percent functional virus-specific CD8^+^ T cells was calculated by dividing the percentage of granzyme B^+^ CD8^+^ T cells by the percentage of tet^+^ cells. **H** Boolean analysis of polyfunctionality of CD8^+^ T cells. Functional markers measured: granzyme B, IFNγ, TNF, perforin, and CD107a. *P < 0.05, **P < 0.005, ****P < 0.0001, one-way ANOVA

To test whether CD8^+^ T cell impairment in aged mice was due to T cell-intrinsic defects or systemic age-dependent dysfunction, we used two complementary approaches. First, using chimeric bone marrow (BM) transplantation, aged and young CD45.2 mice were lethally irradiated and adoptively transplanted with either young or aged CD45.1 T cells along with young B cells and BM from young *Rag1*^−/−^ mice (to reconstitute the non B, non T cell hematopoietic compartment). Engraftment of donor cells with successful ablation of recipient cells was confirmed at 5 weeks post-tranplant (Supp. Figure 5A-C) and was equal in all groups in both transplant models as assessed by donor cell percentages following submandibular venipuncture as well as assessment of cell percentages in the lung and BAL on the day of euthanasia (Supp. Figure 5A-C). There was a significant difference in recipient cell frequencies, but no significant differences in donor cell engraftment (Supp. Figure 5A-C). At six weeks post-transplant, the mice were infected with HMPV and CD8^+^ T cell response analyzed on day 7 p.i. For clarity, the experimental groups are referred to as: age of T cells  ➔ age of host (i.e., young T cells into an aged host = Y_T_➔A_H_) (Fig. 4A). A_T_Y_H_ had a significantly diminished CD8^+^ tet^+^ response compared to Y_T_➔Y_H_ while Y_T_➔A_H_ produced more CD8^+^ tet^+^ cells compared to A_T_➔A_H_ (Fig. 4B-D). Y_T_➔A_H_ mice had more CD8^+^ tet^+^ cells that expressed no inhibitory receptors and fewer CD8^+^ tet^+^ cells that expressed at least one inhibitory receptor compared to A_T_➔A_H_ (Fig. 4E). There was a trend towards increased inflammation measured by higher histopathological score in A_T_➔Y_H_ compared to Y_T_➔A_H_ (Supp. Figure 5D-F).

We next evaluated the function of the transplanted CD8^+^ T cells via the same ex vivo HMPV peptide stimulation as in Fig. 3. Y_T_ CD8^+^ T cells also exhibited increased granzyme B production independent of the age of the host (Fig. 4F). The percentage of functional CD8^+^ HMPV-specific cells was calculated by dividing the percentage of granzyme B^+^ CD8^+^ T cells by the percentage of tet^+^ cells, which was also increased in Y_T_ CD8^+^ T cells (Fig. 4G). Boolean gating of granzyme B, CD107a, IFNγ, TNF, and perforin revealed that Y_T_➔A_H_ mice tended to have more polyfunctional CD8^+^ T cells compared to A_T_➔Y_H_ (Fig. 4H).

**Fig. 5:**
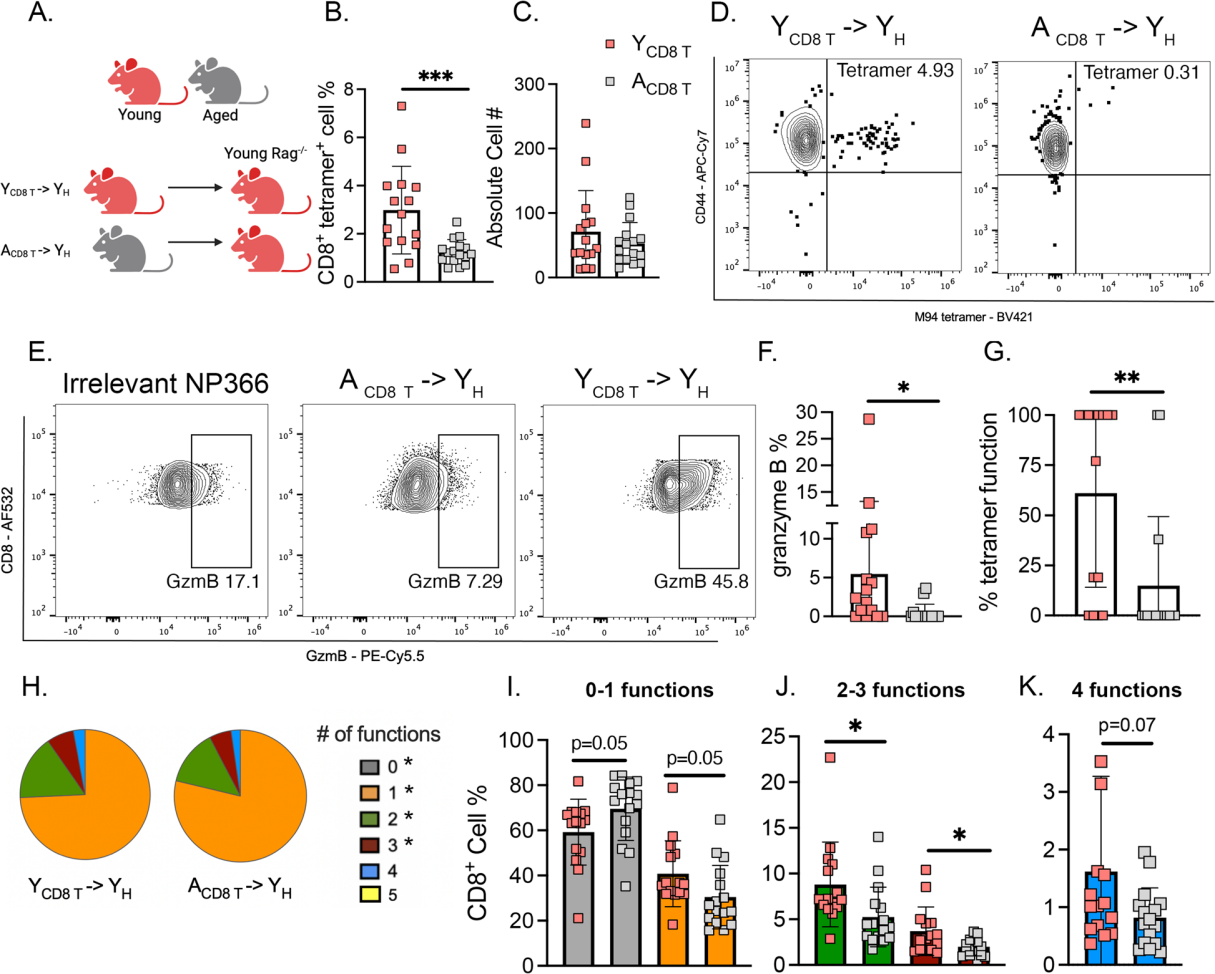
Aged CD8^+^ T cells in *Rag1*^*−/−*^ mice recapitulated aged immune response to HMPV. **A** CD8^+^ T cell adoptive transfer experimental design. Aged or young CD8^+^ T lymphocytes were isolated and adoptively transferred along with young CD4^+^ T and B lymphocytes into young *Rag1*^*−/−*^ recipients. **B**, **C** *Rag1*^*−/−*^ recipients with aged CD8^+^ T lymphocytes (i.e., A_CD8 T_—> Y_H_) had a significantly diminished epitope-specific CD8^+^ T cell response compared to Y_CD8 T_—> Y_H_ control. **D** Representative flow plots of tetramer staining. **E**–**G** A_CD8 T_—> Y_H_ produced less granzyme B and had fewer granzyme B functional CD8^+^ tet^+^ T cells. **H**–**K** A_CD8 T_—> Y_H_ had significantly fewer polyfunctional CD8^+^ T lymphocytes as measured by combinatorial analysis of cytokines/markers of degranulation and SPICE analysis. The percentage of functional virus-specific CD8^+^ tetramer^+^ cells was calculated by dividing the percentage of granzyme B^+^ CD8^+^ T cells by the percentage of tet^+^ cells. Functional markers measured: granzyme B, IFNγ, TNF, perforin, and CD107a. *P < 0.05, **P < 0.01, ***P < 0.005, unpaired t-test or one-way ANOVA

Our second approach to test whether the age-dependent CD8^+^ T cell defects were cell-intrinsic involved adoptive transfer of purified CD8^+^ T cells from young or aged donors into a young *Rag1*^*−/−*^ host, which avoided any potential effects of irradiation and BM reconstitution (Fig. 5A). Similar reduction in the percentage of epitope-specific CD8^+^ T cells was noted in the adoptive transfer model of A_CD8 T_➔Y_H_ which had a diminished CD8^+^ tet^+^ response compared to Y_CD8 T_ ➔Y_H_ (Fig. 5B-D). Aged CD8^+^ T cells transferred to young *Rag1*^*−/−*^ recipients also displayed significantly reduced granzyme B production (Fig. 5E, F), decreased percent functional granzyme B^+^ CD8^+^ T cells (Fig. 5G), and fewer polyfunctional CD8^+^ T cells (Fig. 5H-K). Collectively, these findings from complementary experimental approaches indicate a cell-intrinsic age-related defect in CD8^+^ T cells leading to impaired generation and diminished functionality of HMPV-specific CD8^+^ T cells.

### Aged CD8^+^ T cells exhibit a terminally exhausted phenotype resistant to reinvigoration by PD-1 blockade and 4-1BB treatment

**Fig. 6:**
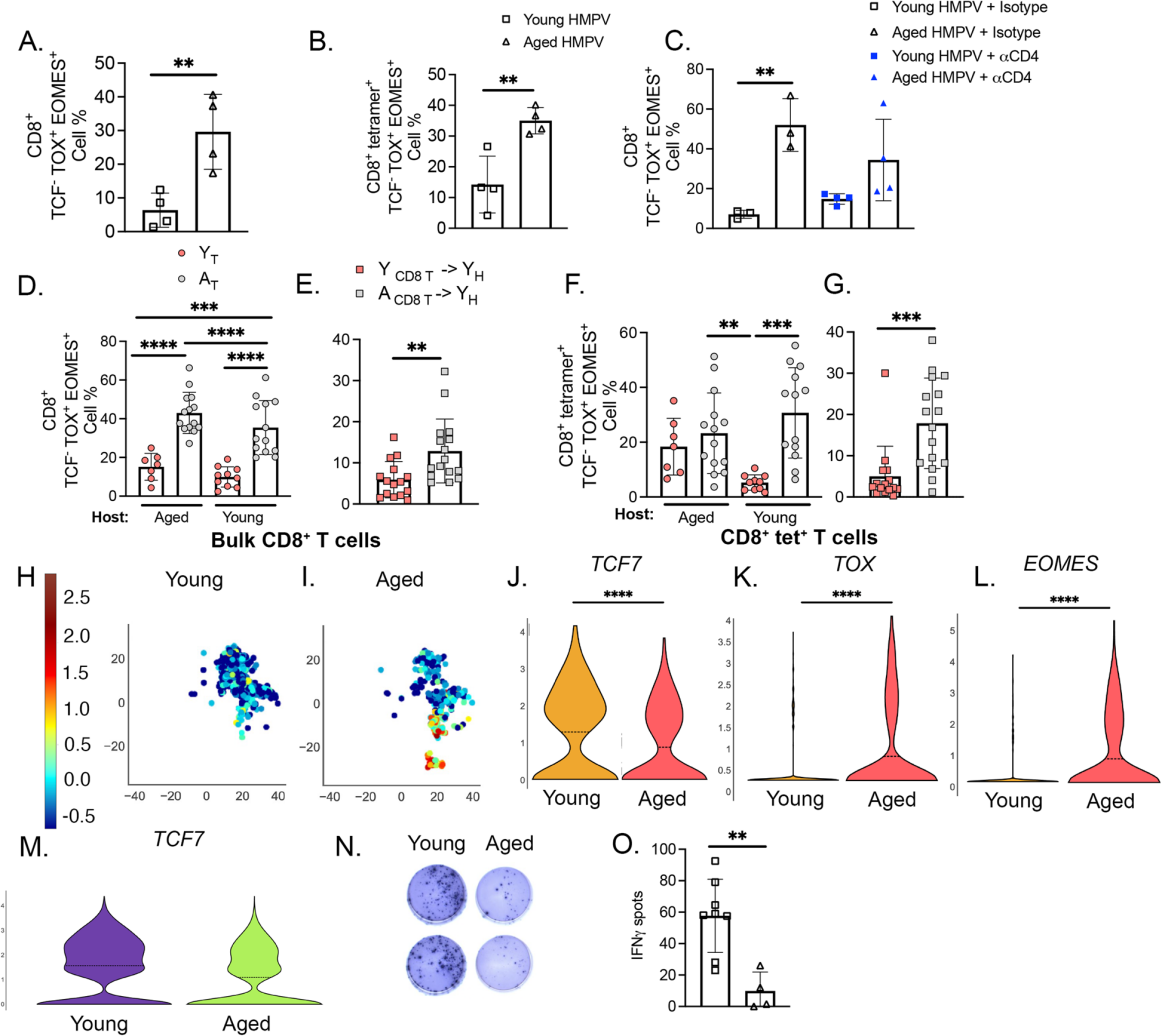
Aged CD8^+^ T cells exhibit terminally exhausted phenotype. **A**, **B** Aged mice at day 7 p.i. had expanded population of terminally exhausted CD8^+^ T cells (T_EX_) in both bulk CD8^+^ T cells and CD8^+^ N11 tet^+^ T lymphocytes. T_EX_ CD8^+^ T cells are defined as TCF1/7^−^ EOMES^+^ TOX^+^. **C** Aged CD4 depleted mice expressed similar levels of TOX and EOMES on CD8s compared to isotype treated mice. Young CD4 depleted mice did have a trend towards increased expression of TOX and EOMES on CD8s. **D** &** F** A_T_—> Y_H_ accumulated T_EX_ bulk and HMPV-specific CD8^+^ T lymphocytes to a similar degree as A_T_—> A_H_ control while Y_T_—> A_H_ and Y_T_- > Y_H_ had reduced T_EX_ populations. **E** &** G** A similar T_EX_ CD8^+^ population in both bulk and epitope-specific CD8^+^ T lymphocytes was seen in the A_CD8 T_—> Y_H_ adoptive transfer model. **H** & **I** scRNAseq on naive young and aged mice revealed similar expression patterns of TOX and EOMES in CD8^+^ T cells from aged lung. **(J-L)** Violin plots of TCF7, TOX, and EOMES expression, respectively, in murine lung CD8^+^ T cells in both age groups. **M** Violin plot of TCF7 expression in PBMCs from healthy aged and young humans. **N** Representative IFN ELISpot image. **O** IFN spot number significantly decreased in aged mice. *P < 0.05, **P < 0.01, ***P < 0.001, ****P < 0.0001, unpaired t-test or one-way ANOVA

Given the high degree of functional impairment and expression of numerous cell surface inhibitory receptors, we hypothesized that age-related terminal exhaustion was a contributing factor to the impaired function of aged CD8^+^ T cells, even when transplanted into young mice. To test this, we first measured baseline expression of *EOMES*, *TOX*, and *TCF1/7* in HMPV-infected aged and young mice since these transcription factors are key in characterizing terminal exhaustion [16, 27]. There was a striking increase in exhausted TCF1/7^−^ EOMES^+^ TOX^+^ bulk CD8^+^ T cells (T_EX_) (Fig. 6A) and an increase toward more N11 epitope-specific TCF1/7^−^ EOMES^+^ TOX^+^ CD8^+^ T cells in aged versus young mice at day 7 p.i. (Fig. 6B). Representative gating is shown in (Supp. Figure 6). To determine if this phenotype represented an exhausted CD8^+^ T cell as opposed to a transiently impaired state, we depleted CD4^+^ T cells from aged and young HMPV-infected mice. Previous studies have shown that CD4^+^ T cells prevent CD8^+^ T cell exhaustion [28, 29]. Therefore, depleting CD4^+^ T cells would promote CD8^+^ T cell exhaustion similar to chronic antigen stimulation models (i.e. chronic viral infection, cancer). Aged CD4 depleted mice had a similar population of TOX^+^ EOMES^+^ CD8^+^ T cells as isotype control treated mice of the same age (Fig. 6C). These findings indicate that this population of T_EX_ CD8^+^ represents a CD8-intrinsic exhausted state.

We next examined CD8^+^ T_EX_ cells in the BM chimera transplanted groups to determine whether the aged CD8^+^ T_EX_ phenotype was dependent upon the age of the host. We saw a marked increase in the T_EX_ population in the A_T_➔Y_H_ mice compared to the Y_T_➔Y_H_ mice with a similar striking increase in T_EX_ in A_T_➔A_H_ compared to Y_T_➔A_H_ in both bulk and epitope-specific CD8^+^ T cells (Fig. 6D, F). The adoptive transfer model of aged or young CD8^+^ T cells into *Rag1*^*−/−*^ mice also demonstrated an increase in T_EX_ in A_CD8 T_➔Y_H_ in both bulk and tetramer^+^ CD8^+^ T cells (Fig. 6E, G). Taken together, these findings suggest that a key cell-intrinsic driver of aged CD8^+^ T cell dysfunction is a terminal exhaustion which cannot be reversed by transplanting aged CD8^+^ T cells into a young host.

Using publicly available datasets from a prior study [16] in which scRNAseq was performed on lungs from naïve, uninfected young and aged mice, as well as on PBMCs from young and aged healthy humans, we found a similar T_EX_ CD8^+^ population in aged murine lung (Fig. 6H & I). Aged CD8^+^ T cells from both uninfected mice and humans had decreased *TCF1/7* expression (Fig. 6J; M) while aged CD8^+^ T cells from mice also upregulated *TOX* (Fig. 6K) and *EOMES* (Fig. 6L). Furthermore, in our model, aged lung lymphocytes, following ex vivo Class I peptide stimulation, produced significantly less IFNγ as compared to young lung lymphocytes (Fig. 6N & O). Taken together, these findings support our data identifying a T_EX_ CD8^+^ T cell population in aged mice that is functionally impaired and unable to be reinvigorated with transplantation into a young host.

We next sought to uncover whether aged CD8^+^ T cell exhaustion could be reversed and function restored by existing therapeutics. To this end, we administered a PD-1 blocking antibody (αPD-1), a 4-1BB agonist, or both to aged mice and then infected them with HMPV. These approaches have previously been shown to rejuvenate exhausted T cells in cancer models [30, 31], and PD-1:PD-L blockade restored CD8^+^ T cell function in HMPV-infected young adult mice [11, 13]. None have been described in the setting of HMPV infection of aged individuals. Using our previously established ELISpot assay for assessing response to inhibitory receptor blockade [11], we found that lung lymphocytes from aged mice produced less IFNγ compared to young mice (Supp. Figure 7A-C) and this response failed to improve with PD-1 blockade, 4-1BB treatment, or a combination in aged mice (Supp. Figure 7A-C). These data indicate that aged CD8^+^ T cells are resistant to functional reinvigoration with checkpoint inhibitors that have shown clinical efficacy in humans and mice for reversing T cell exhaustion.

### Influenza-infected aged mice had impaired CD8^+^ T cell response and accumulated terminally exhausted CD8^+^ T cells

**Fig. 7:**
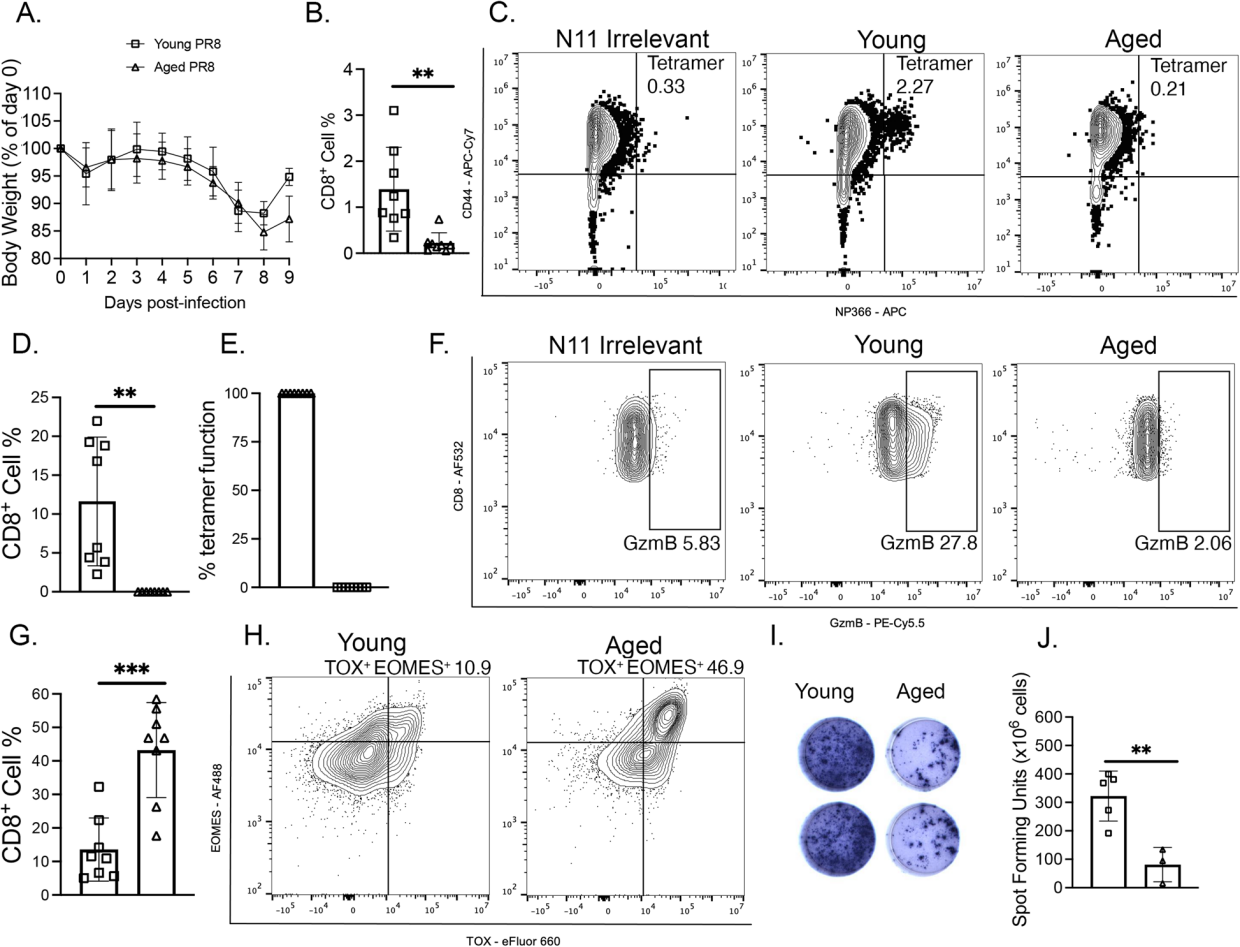
Influenza infected aged mice had impaired and terminally exhausted CD8^+^ T cells. **A** Aged infected mice tended to lose more weight by day 9 p.i. **B** Aged mice produced fewer tet^+^ CD8^+^ T cells at day 7 p.i. **C** Representative flow plots of NP366 flu tetramer staining at day 7 p.i. **D** Aged CD8^+^ T cells failed to produce granzyme B. **E** Aged CD8^+^ T cells had no granzyme B functional tet^+^ T cells. **F** Representative granzyme B staining at day 7 p.i. **G** Aged mice accumulated more TCF^−^ TOX^+^ EOMES^+^ CD8^+^ T cells compared to young infected mice at day 7 p.i. **H** Representative staining of TOX^+^ EOMES^+^ CD8^+^ T cells. **I** Representative images of ELISpot. **J** Aged mice had minimal IFN production, *P < 0.05, **P < 0.01, ***P < 0.005, unpaired t-test

We sought to determine if this intrinsic CD8^+^ T cell terminal exhaustion and functional impairment was generalizable to other respiratory viruses. Aged mice infected with influenza tended to lose more weight by day 8 and 9 p.i. (Fig. 7A) and also produced fewer influenza-specific CD8^+^ T cells (Fig. 7B-C). Aged influenza-infected mice failed to produce granzyme B or have any granzyme B functional tet^+^ CD8^+^ T cells (Fig. 7D-F). There was also a striking accumulation of aged CD8^+^ T cells that were TOX^+^ EOMES^+^ (Fig. 7G-H). Furhermore, aged CD8^+^ T cells were functionally impaired, producing less IFNγ at day 7 p.i. (Fig. 7I & J). These findings underscore that the aged mouse phenotype of functionally impaired, exhausted CD8^+^ T cells is present in response to multiple respiratory viral infections.

## Discussion

Here, we examined the aged CD8^+^ T cell response to HMPV using a newly established aged mouse model. We found that aged mice exhibit enhanced disease and delayed viral clearance compared with young mice. Aged mice also generate fewer lung resident virus-specific T cells, possibly due to reduced precursor frequency or differentiation potential, and those that do develop are functionally impaired. Furthermore, the T cell phenotype in aged mice is intrinsic to the CD8^+^ T cell compartment, with a terminal exhaustion phenotype that was also found in single cell analysis [16] from naïve, uninfected, aged mice and healthy, aged humans, further supporting our findings. Functionally, aged CD8^+^ T cells were resistant to reinvigoration by PD-1 checkpoint inhibitor and 4-1BB treatment. Moreover, this age-related CD8^+^ T cell exhaustion contributes to increased disease severity caused by multiple respiratory viruses including HMPV and influenza.

These findings have important implications for our understanding of severe lower respiratory tract infections in the elderly, which is a leading worldwide cause of morbidity and mortality in this vulnerable population [4, 5]. One previous study reported increased weight loss, enhanced viral burden, and elevated pro-inflammatory cytokine production in 18-month-old HMPV-infected BALB/c mice compared to 6–7 week-old mice [32]. In that report, HMPV-infected aged and young mice had similar numbers of pulmonary CD8^+^ T cells, although antigen specificity and function were not assessed. We developed an aged mouse model that recapitulated severe HMPV illness that is observed in humans. Depletion of CD8^+^ T cells in aged and young mice during infection revealed that CD8^+^ T cells are not solely responsible for weight loss and viral clearance since we observed no difference in weight loss between isotype treated and CD8 depleted groups of either age with minimal difference in viral clearance. These findings are consistent with a previous study showing the dual role of CD8^+^ and CD4^+^ T cells in viral clearance [26]. In our study, we focused on the CD8^+^ T cell response to HMPV, acknowledging that viral clearance and weight loss during infection are multifactorial processes. We did find that aged CD8^+^ T cells are intrinsically impaired, which contributed to a diminished adaptive immune response and more severe disease. CD8^+^ T cell dysfunction has also been observed in other lower respiratory infections in elderly humans, including influenza [23] and COVID-19 [33], supporting our findings.

We found that aged mice produced significantly fewer HMPV epitope-specific CD8^+^ T cells in the lung and BAL. This has been reported in aged mice infected with influenza [21], but has not been shown for HMPV previously. We determined that this decrease in epitope-specific CD8^+^ T cells was not due to impaired migration, as there was no epitope-specific aged CD8^+^ T cell accumulation in the draining lymph nodes or spleen. Aged uninfected mice had a diminished pool of naïve CD44^−^ CD62L^+^ CD8^+^ T cells, which may lead to the reduced number of naïve CD8^+^ T cells and the limited capacity of these naïve CD8^+^ T cells to differentiate into T_EM_. This phenomenon of depleted naïve T cells in aged antigen-experienced mice, limiting the antigen-specific precursor repertoire, has been described for both CD4^+^ and CD8^+^ T cells [34, 35]. A previous study using vaccina virus characterized virus-specific precursors and determined their necessity to confer protective T cell immunity [36]. Thus, the important finding of fewer epitope-specific precursor CD8^+^ T cells in our model in uninfected aged mice further supports and underscores an age-dependent diminished virus-specific CD8^+^ T cell response to HMPV infection [30, 37].

Virus-specific CD8^+^ T cells in aged mice infected with HMPV co-expressed more inhibitory receptors and were less polyfunctional compared to those from young mice. Notably, aged CD8^+^ T cells had a striking decrease in granzyme B production, a phenomenon that has been described for other aged mouse models using single cell RNA sequencing analysis [16] as well as in elderly humans infected with COVID-19 [33]. Our results show that HMPV-infected aged mice produced virus-specific CD8^+^ T cells that were fewer in number, expressed more inhibitory receptors, and were deficient in granzyme B production and IFNγ secretion, thus explaining many features of the aged HMPV phenotype.

We used two complementary models, syngeneic transplant and adoptive transfer, to determine whether this aged CD8^+^ T cell phenotype was due to a cell-intrinsic or host-dependent extrinsic cause. With both approaches, we observed that aged CD8^+^ T cells exhibited decreased function regardless of whether the recipient host was young or aged. Similarly, young CD8^+^ T cells did not show any impairment when transplanted into an aged host. Previous studies support these findings by showing that transplants of aged bone marrow or aged CD8^+^ T cells alone into a young host is insufficient for the aged immune cells to be rejuvenated [16, 38]. Collectively, these results indicate that the aged CD8^+^ T cell phenotype in our model in response to HMPV infection originates primarily from a cell-intrinsic source. Of note, transfer of young CD8^+^ T cells into aged mice by either transplant approach did not fully recapitulate all immunologic, virologic, and pathologic features of T cells in young mice during primary HMPV infection. This could be due to immunologic alteration due to the irradiation and transfer procedures, the semi-permissive nature of murine models for human pathogens, or a contribution of aging in the somatic cell compartment. Ongoing work is exploring the last possibility.

Since HMPV causes an acute infection, we thought it unlikely that HMPV disease was causing classical CD8^+^ T cell exhaustion described in chronic infection [39] and cancer models [30, 40]. Rather, the aged microenvironment may promote a terminal exhaustion-like state in CD8^+^ T cells, which then impairs the immune response when the aged host is exposed to HMPV. Previous studies have shown a baseline increase in *EOMES* and *TOX* with a decrease in *TCF1/7* on CD8^+^ T cells in both uninfected aged mice [16] and aged humans [25, 41]. In our study, we found that aged mice, both uninfected and infected, accumulated a large population of TCF1/7^−^ EOMES^+^ TOX^+^ CD8^+^ T cells (i.e., T_EX_) compared to young mice, which correlated with a loss of IFNγ production in aged mice. This finding was recapitulated in the transplant and adoptive transfer models, where aged bulk and, importantly, virus-specific CD8^+^ T cells, retained this T_EX_ phenotype despite being transferred into a young host. Conversely, young CD8^+^ T cells did not acquire a T_EX_ phenotype when transferred into an aged host. When aged and young HMPV-infected mice were depleted of CD4 T cells to mimic CD8^+^ T cell exhaustion, there was no significant increase in the population of TCF1/7^−^ TOX^+^ EOMES^+^ CD8^+^ T cells between isotype and CD4^+^ depleted aged mice, indicating that the CD8^+^ T_EX_ phenotype observed during HMPV infection was canonical exhaustion. Importantly, we observed an increase, although not statistically significant in TCF1/7^−^ TOX^+^ EOMES^+^ CD8^+^ T cells in young CD4-depleted HMPV-infected mice compared to young isotype control. We may not have seen a robust increase in TCF1/7^−^ TOX^+^ EOMES^+^ CD8^+^ T cells in young CD4-depleted mice because our model is an acute viral infection while other studies [28, 29] used CD4 depletion as a syngergistic approach along with a cancer or chronic infection model to promote CD8^+^ T cell exhaustion. Overall, these findings suggest an age-dependent progression of CD8^+^ T cells to a terminally exhausted phenotype, which limits antiviral CD8^+^ T cell responses.

Terminal exhaustion has been partially reversible in other models of T cell exhaustion in which there is chronic antigen stimulation or a tumor microenvironment. In mice chronically infected with lymphocytic choriomeningitis (LCMV), TCF1/7^+^ CD8^+^ T cells were identified as the CD8^+^ T cell subset responsive to PD-1 blockade [42]. PD-1 blockade and 4-1BB treatment were also able to reverse CD8^+^ T cell exhaustion in cancer models [30, 43]. In our model, we identified a subset of CD8^+^ T cells in the aged host that possess transcriptional markers resembling T_EX_ found in these chronic infection and cancer models. However, unlike cancer [37, 43] and chronic viral infection models [42], attempts to reverse the T_EX_ phenotype in aged CD8^+^ T cells using PD-1 blockade and 4-1BB treatment were not successful. Taken together, the data from the current experiments suggests that age-dependent changes in CD8^+^ T cells related to viral infection may, indeed, be irreversible and thus represent a true terminal exhaustion phenotype similar to chronic antigen stimulation models. These data point towards a need to identify other targets or novel approaches to therapy. Future studies will focus on understanding why checkpoint inhibitors fail to improve the immune response in the aged host and finding additional treatments that can be used.

## Conclusions

Here we present evidence that a major contributor to severe HMPV disease in older adults is due to age-dependent, cell-intrinsic CD8^+^ T cell dysfunction. Aged mice exhibited a diminished virus-specific response to both HMPV and influenza with decreased functionality, which led to clinical and virologic consequences. We identified an accumulation of terminally exhausted CD8^+^ T cells in the aged host during both HMPV and influenza, whose function was unable to be meaningfully restored with checkpoint inhibitor treatment. These findings have important implications for severe respiratory virus infections among elderly individuals, as well as older adults with cancer receiving checkpoint inhibitor therapy who acquire respiratory viral infections. Additionally, these findings provide further insight into the pathophysiology of severe respiratory viral infection among older adults and our model builds a foundation for future studies to improve the aged immune response to respiratory viruses and vaccines.

## Methods

### Mice and viral infection

C57BL/6 (B6), congenic CD45.1, and *Rag1*^−/−^ mice were purchased from The Jackson Laboratory. All animals were bred and maintained in specific pathogen-free conditions in accordance with the University of Pittsburgh Institutional Animal Care and Use Committee. 6-7wks and 70-71wks female animals were used in all experiments. HMPV (pathogeneic clinical strain TN/94–49, genotype A2) was grown and titered in LLC-MK2 cells as previously described [44]. In select experiments, influenza virus strain A/34/PR/8 (PR8) was grown in MDCK cells and titered on LLC-MK2 cells as in [10]. For all experiments, mice were anesthetized with isoflurane in a heated chamber and infected intratracheally with 2.0 × 10^6^ PFU HMPV or 500 PFU PR8 in 100-$\upmu$ L volume. Mock-infected mice were inoculated with the same volume of sterile PBS. Viral titers for HMPV infected mice were measured by plaque assay as previously described [44, 45]. Clinical scoring of mice was performed by at least two individuals independently with one point given for each of the following signs: hunched, huddled, rapid breathing, lethargy, or ruffled fur.

### Bronchoalveolar lavage (BAL)

BAL was harvested as in described [46]. Briefly, mice were euthanized and their trachea exposed, cannulated with a blunt tipped syringe needle, and secured in place using suture. Lungs were flushed with 1 mL BAL buffer (sterile PBS, 0.5% Fetal Bovine Serum (FBS), 1:250 0.5 M EDTA) at least four times. BAL was spun down at 500xg for 10 min. Supernatant was collected for Luminex. 1 mL of ACK lysis buffer was added to the cell pellet. After 1 min, 1 mL of RPMI/10% FBS was added and spun down at 500xg for 5 min. Cells were resuspended in RPMI/10% FBS and used for flow cytometry staining.

### Histopathologic score

10% formalin was injected into a section of the lower left lung lobe and stored in 10% formalin in histology cassettes (Fisher Scientific B851000WH). Tissue sections were stained with H&E by the UPMC Children’s Hospital of Pittsburgh Histology Core and slides were imaged and scored at 200X magnification. Scoring criteria per field included: 0: no inflammation; 1: < 25% inflammation; 2: 25–50% inflammation; 3: 50–75% inflammation; 4: > 75% inflammation. To generate the histopathologic score, the score for each sample was added and divided by the total number of fields analyzed.

### Antibody treatment

#### For CD8^+^ T cell depletion

One day prior to infection, aged and young B6 mice were treated with 300 μg in sterile PBS of αCD8 (BioXCell BE0061) or rat isotype control (BioXCell BE0090) via intraperitoneal injections. Mice were injected with 150 μg of αCD8 or rat isotype control every other day post-infection.

### IFNγ ELISpot assay

ELISpot assay and analysis was performed as previously described [10, 47] adding HMPV N_11-18_ peptide. Influenza NP366 peptide served as control.

### Flow cytometry staining

#### Single cell suspension

Mice were euthanized and the right lung harvested. The lung was cut into 2 mm segments using scissors in Eppendorf tubes. Lung tissue was resuspended in RPMI/10% FBS in tissue culture tubes and incubated for 1 h at 37 °C with DNAse and collagenase. After enzymatic digestion, the lung was filtered through 70 μm filters, spun down at 1500 rpm for 5 min, and the pellet resuspended in 2 mL ACK Lysis Buffer (Gibco A10492-01) for 1 min. 10 mL RPMI/10%FBS was added after ACK Lysis and cells were spun down at 1500 rpm for 5 min. Cells then underwent either tetramer staining or ex vivo peptide stimulation.

#### Tetramer staining

Cells were incubated with FACS/desatinib for 30 min before adding APC conjugated N_11-18_ or BV421 conjugated M_94-102_ tetramers 1:200 in FACS/desatinib for 90 min. Cells were then spun down at 1500 rpm for 3 min and washed 1 × with FACS buffer.

#### Ex vivo* peptide stimulation*

100μL of cells were added to a flat-bottom 96-well tissue culture plate. The following was added to cells: 100μL of 200 μM N11 HMPV peptide or NP366 for irrelevant control diluted 1:10 in RPMI/10% FBS, 6μL CD107a-PE, and 22μL BFA (BD Cat. #51-2301KZ)/Monensin (BD Cat. #2092KZ). In addition, 1:1000 PMA/ionomycin instead of peptide was added to one aliquot of cells for a positive control. Cells were incubated for 5 h at 37 °C.

*For both conditions: *After either tetramer staining or peptide stimulation, cells were stained with Live/Dead dye 1:1000 in PBS for 12 min, washed 1 × with PBS, and blocked with αCD16/32 Fc block (Tonbo Biosciences Cat. #70–0161-M001) 1:100 in FACS buffer for 10 min. For surface staining, cells were stained with surface antibody 1:100 in BD Brilliant Stain Buffer (BD Cat. #566,349) buffer for 30 min at 4 °C. Cells were spun down at 1500 rpm for 3 min and washed 1 × with FACS buffer.

#### Intracellular cytokine staining

After cells were stained for surface antibodies, cells were fixed for 30 min with eBioscience™ Foxp3/Transcription Factor Staining Buffer Set (ThermoFisher 00–5523-00) at 4 °C, spun down at 1640 rpm for 3 min, washed 1 × with Foxp3 Fix/Perm Buffer, and stained with 6uL/antibody in Foxp3 Fix/Perm Buffer for 1 h at 4 °C. Cells were spun down at 1640 rpm for 3 min, washed 1 × with FACS buffer, resuspended in FACS buffer, and stored in the dark at 4 °C until they were analyzed on the Cytek® Aurora multispectral flow cytometer.

#### Intracellular transcription factor staining

For transcription factor staining, cells were fixed for 18 h in Foxp3 Fix/Perm at 4 °C. After fix/perm, cells were washed 1 × with Foxp3 Fix/Perm Buffer and stained with 2.5μL antibody in Foxp3 Fix/Perm Buffer for 1 h at 4 °C.

After intracellular staining, cells were spun down at 1640 rpm for 3 min, washed 1 × with FACS buffer, resuspended in FACS buffer with 100μL BioLegend Precision Count Beads™ (BioLegend Cat. #424,902) and run on the Cytek® Aurora multispectral flow cytometer. A full list of antibodies used in all experiments is shown in Supplemental Table 1. Fluorescence minus one (FMO) controls were used for all inhibitory receptors and transcription factors. For HMPV tetramer staining, Flu NP366-APC and HLA-B-35:01-BV421 tetramers were used as the irrelevant controls. Any irrelevant tetramer background staining was subtracted from the final tetramer frequency. Unstained cells from each experiment were fixed for 20 min in 2% PFA and used on the flow cytometer to minimize autofluorescence. Data analysis was performed with Flowjo(v10.8.1). Boolean gating in FlowJo was used to assess inhibitory receptor and functional cytokine co-expression. Patterns were visualized using the SPICE program (NIAID).

### Bone marrow transplant

#### Irradiation

The day prior to transplant, recipient mice were conditioned with 10–11 cGy total body irradiation in two split doses four hours apart from an X-ray source (MultiRad 350, Precision X-Ray Irradiation). Irradiated mice were placed on an immunocompromised rack in animal facility, given sterile autoclaved water, and supplied with irradiated food.

#### BM single cell suspension

Femur and tibia from *Rag1*^*−/−*^ mice were harvested, tissue removed, and clean cuts made at either bone end. Using a 25G needle, marrow was flushed from the bone into a conical tube using D-10 media (10% FBS, 1% Pen Strep antibiotics, 1% L-glutamine, 1% MEM Non-Essential Amino Acids, 0.1% 50 mM β-mercaptoethanol). BM was spun down at 350xg for 5 min and cell pellet was filtered through 70 μm strainer, washed 2 × with 5 mL sterile PBS, and counted on BD Accuri™ cytometer.

#### B and T cell magnetic column selection

For B and T lymphocyte magnetic column selection, spleen and lymph nodes (inguinal, peritoneal, and submandibular) were collected from B6 and congenic CD45.1 donor mice either 6-7wks or 70-71wks. All lymph nodes and each spleen per mouse were passed through 70um strainer, spun down at 350xg for 5 min, pooled together through a 40um strainer, and spun down again at 350xg for 5 min. The spleen/lymph node single cell suspension were combined from two mice and resuspended in 900μL aMACs buffer at which point cells were incubated with CD90.2 microbeads or CD19-biotin and biotin labeled microbeads and run over LS column (Miltenyi 130–042-401) per Miltenyi instructions. Plunge was collected from the columns, spun down at 350xg for 5 min, and cell pellet was washed with 5 mL sterile 1XPBS × 2. Cells were counted on BD Accuri™ cytometer.

#### Tail vein injections

1 × 10^7^ T, B, and *Rag1*^*−/−*^ BM cells were resuspended per ml of sterile PBS, followed by injection of 200μL of cells (2 × 10^6^ of each cell type) into irradiated recipient mice via tail vein injection.

### CD4^+^ and CD8^+^ T cell adoptive transfer

CD4 and CD8 biotinylated antibodies along with anti-biotin microbeads were used to positively select CD4 and CD8 subsets of T cells for use in combination experiments. 1 × 10^6^ CD4 and 1 × 10^6^ CD8 T cells were combined per mouse, with the remainder of the cell populations as outlined above.

### scRNAseq data analysis

CD8^+^ T cell scRNAseq dataset from lungs of mice and humans was kindly shared with us from Maxim Artyomov: https://www.synapse.org/#!Synapse:syn22255433/wiki/604556 and was generated as described in [16].

### Statistical analysis

Data analysis was performed using Prism version 9.0 (GraphPad Software). Comparisons between 2 groups were performed using an unpaired 2-tailed Student’s *t* test. Multiple group comparisons were performed using a 1-way or 2-way ANOVA as appropriate. A *P* value less than 0.05 was considered significant. Error bars in each graph represent SEM.

## Supplementary Information

**Additional file 1: Supplemental Table 1.**

**Additional file 2: Supplemental Figure 1.** There was an increase in CD4+ T cells with CD8+ depletion. (A) Aged and young mice were treated with 300g CD8 or rat isotype control Ab one day prior to infection and 150g every other day post-infection via intraperitoneal injection. Representative flow plots from isotype control and CD8 treated on day 5 p.i. (B & C) There was an increase in CD4+ T cells by cell percent in both age groups at day 7 p.i. and an increase in CD4+ absolute cell number in aged CD8+ depleted mice. *P<0.05, **P<0.01, one-way ANOVA.

**Additional file 3: Supplemental Figure 2.** The impaired tetramer response in aged mice was not epitope specific. (A) Aged infected mice had decreased CD8+ M94 tetramer+ cells in lung (shaded bars) and BAL (open bars) compared to young infected mice at day 7 post-infection. (B) Absolute cell number of M94+ CD8+ T cells. (C) Representative flow plots of tetramer staining on activated CD44 CD8+ T cells. *P<0.05, unpaired t-test.

**Additional file 4: Supplemental Figure 3.** Similar CD8+ counts and minimal tet+ cells in secondary lymphoid organs. (A) There was no difference between age groups in total CD8+ T cells in either lung or BAL at day 7 post-infection. (B & C) Aged mice tended to have more HMPV-specific CD8+ T cells by cell percent in the draining lymph nodes and spleen, but there was no difference between the two age groups in absolute cell number. (D) Representative flow plots aged and young infected lymph nodes on day 7 post-infection with influenza NP366 irrelevant tetramer as a control.

**Additional file 5: Supplemental Figure 4.** Aged mice had fewer CD44- CD62L+ CD8+ T cells in lung. (A & B) Uninfected aged mice at baseline had significantly fewer naïve CD8+ CD44- CD62L+ T cells (TN) in the lung compared to uninfected young mice. Upon infection, young mice had a robust increase in CD8+ CD44+ CD62L- effector memory cells (TEM) while aged mice had only a modest increase. Bar graphs showing the composition of CD44 and CD62L expression in CD8+ T cells shown in B with raw data points shown in B. (C) Representative flow plots of CD44 and CD62L expression shown for each age group and infection status.

**Additional file 6: Supplemental Figure 5.** No significant differences in donor T cell engraftment in transplant models. (A-C) Mice were bled by submandibular venipuncture 5 weeks post-irradiation and transplant. Lymphocytes were stained with congenic markers CD45.1 and CD45.2 to determine donor cell engraftment. Graph shows the relative frequencies of donor and recipient CD8+ T lymphocytes from peripheral blood, BAL, and lung, respectively. There was a difference in recipient cells remaining in aged and young hosts, but no signficant differences in donor T cell engraftment. (D) AT -> YH tended to have a higher histopathology score compared to YT -> AH at day 7 p.i. (E & F) Representative histology shown. ****P<0.0001, unpaired t-test.

**Additional file 7: Supplemental Figure 6.** Representative flow plots of TEX CD8+ T cells.

**Additional file 8: Supplemental Figure 7.** PD-1 blockade, 4-1BB treatment does not improve aged CD8+ T cell function. Ex vivo peptide stimulation of aged or young lung lymphocytes were isolated day 7 p.i. were treated with isotype control antibody, PD-1 blockade, 4-1BB costimulation, or a combination. (A-C) IFNγ spot number was increased and spot size significantly increased in young T lymphocytes treated with 4-1BB, PD-1, and a combination while aged T lymphocytes did not show an improvement in function with any treatment. *P<0.05, ***P<0.001, ****P<0.0001, one-way ANOVA.

## Data Availability

The datasets used and/or analyzed during the current study are available from the corresponding author on reasonable request.
