# Supplementary material for: Terminally exhausted CD8+ T cells contribute to age-dependent severity of respiratory virus infection
Source: Immun Ageing. 2023 Aug 1;20:40. doi: 10.1186/s12979-023-00365-5 (PMC10391960; doi:10.1186/s12979-023-00365-5)
Supplement: Supplementary file 1 — Additional file 1: Supplemental Table 1. [file 12979_2023_365_MOESM1_ESM.pdf]

Supplemental Table 1.

**Cell Marker**	**Fluorophore**	**Species**	**Catalog Number**	**Clone**
Both plates				
CD19	BV785	rat	115543	6D5
CD3e	BUV395	hamster	565992	145-2C11
CD4	AF700	rat	100536	RM4-5
CD44	APC-Cy7	rat	560568	IM7
CD62L	BUV563	rat	741230	MEL-14
CD8a	AF532	rat	58-0081-80	53-6.7
Tetramer plate only				
Foxp3	PerCP-Cy5.5	rat	45-5773-82	FJK-16s
T-bet	PE	mouse	644809	4B10
GATA-3	BV711	mouse	565449	L50-823
Rorγt	AF647	mouse	562682	Q31-378
EOMES	AF488	mouse	53-4875-82	Dan11mag
TOX	eFluor™660	mouse	50-6502-82	TXRX10
TCF-7/TCF-1	R718	mouse	567587	S33-966
BCL6	BUV661	mouse	568062	K112-91
PD-1 (CD279)	PE-Cy7	rat	109110	RMP1-30
TIM-3 (CD366)	BV605	rat	119721	RMT3-23
LAG-3 (CD223)	BUV805	rat	748540	C9B7W
2B4 (CD244.1)	BUV737	rat	749155	C9.1
HMPV M94 Class I tetramer	BV421	--	From NIH	--
HMPV N11 Class I tetramer	APC	--	From NIH	--
Peptide stimulation plate only				
Perforin	FITC	rat	11-9392-82	eBioOMAK-D
Granzyme B	PE Cy5.5	Rat	35-8898-80	NGZB
TNFα	BUV661	rat	750025	MIH44
IFNγ	BV650	rat	505831	XMG1.2
CD107a (LAMP1)	PE	rat	121611	1D4B
IL-2	APC	rat	503810	JES6-5H4
