# Supplementary figures and images for: Terminally exhausted CD8+ T cells contribute to age-dependent severity of respiratory virus infection
Source: Immun Ageing. 2023 Aug 1;20:40. doi: 10.1186/s12979-023-00365-5 (PMC10391960; doi:10.1186/s12979-023-00365-5)

A.

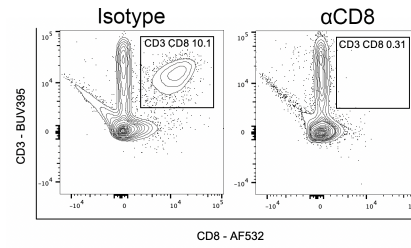

B.

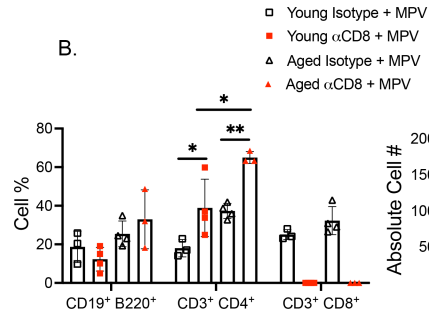

C.

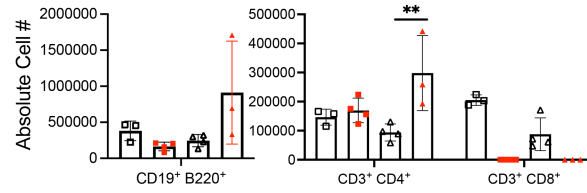

Supplement: Supplementary file 2 — Additional file 2: Supplemental Figure 1. There was an increase in CD4+ T cells with CD8+ depletion. (A) Aged and young mice were treated with 300g CD8 or rat isotype control Ab one day prior to infection and 150g every other day post-infection via intraperitoneal injection. Representative flow plots from isotype control and CD8 treated on day 5 p.i. (B & C) There was an increase in CD4+ T cells by cell percent in both age groups at day 7 p.i. and an increase in CD4+ absolute cell number in aged CD8+ depleted mice. *P<0.05, **P<0.01, one-way ANOVA. [file 12979_2023_365_MOESM2_ESM.pdf]

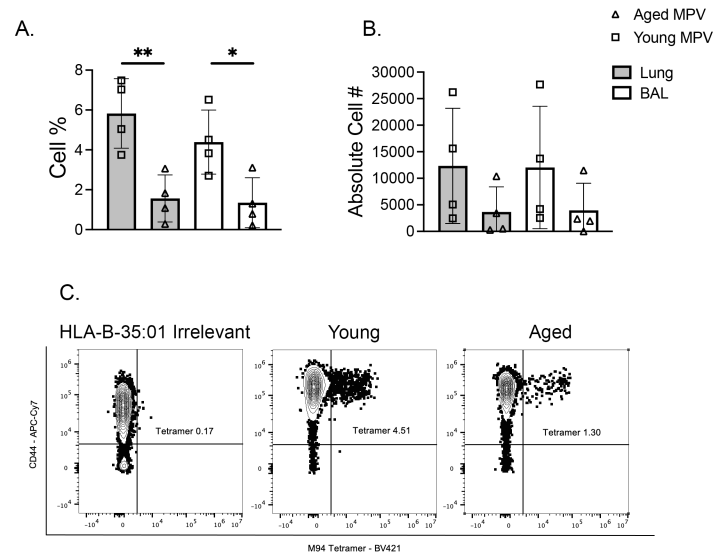

Supplement: Supplementary file 3 — Additional file 3: Supplemental Figure 2. The impaired tetramer response in aged mice was not epitope specific. (A) Aged infected mice had decreased CD8+ M94 tetramer+ cells in lung (shaded bars) and BAL (open bars) compared to young infected mice at day 7 post-infection. (B) Absolute cell number of M94+ CD8+ T cells. (C) Representative flow plots of tetramer staining on activated CD44 CD8+ T cells. *P<0.05, unpaired t-test. [file 12979_2023_365_MOESM3_ESM.pdf]

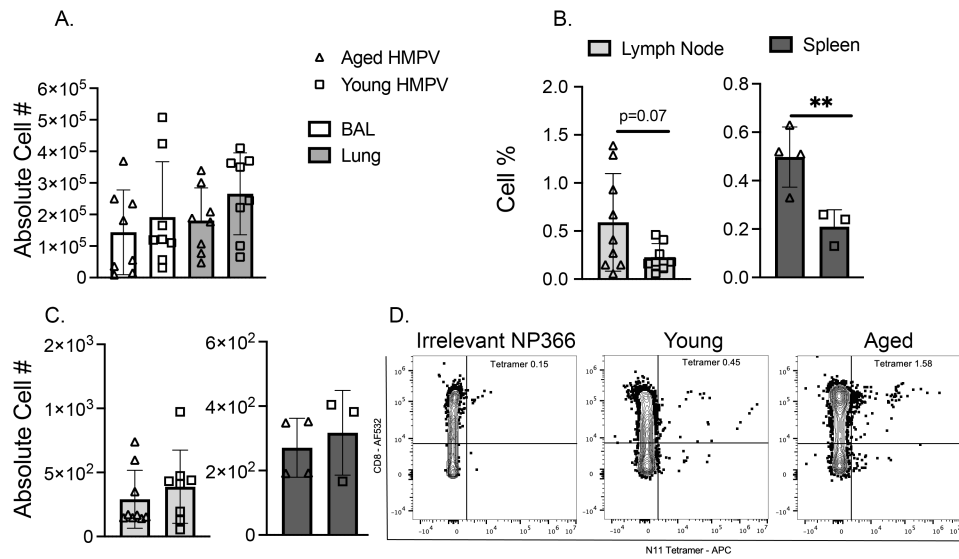

Supplement: Supplementary file 4 — Additional file 4: Supplemental Figure 3. Similar CD8+ counts and minimal tet+ cells in secondary lymphoid organs. (A) There was no difference between age groups in total CD8+ T cells in either lung or BAL at day 7 post-infection. (B & C) Aged mice tended to have more HMPV-specific CD8+ T cells by cell percent in the draining lymph nodes and spleen, but there was no difference between the two age groups in absolute cell number. (D) Representative flow plots aged and young infected lymph nodes on day 7 post-infection with influenza NP366 irrelevant tetramer as a control. [file 12979_2023_365_MOESM4_ESM.pdf]

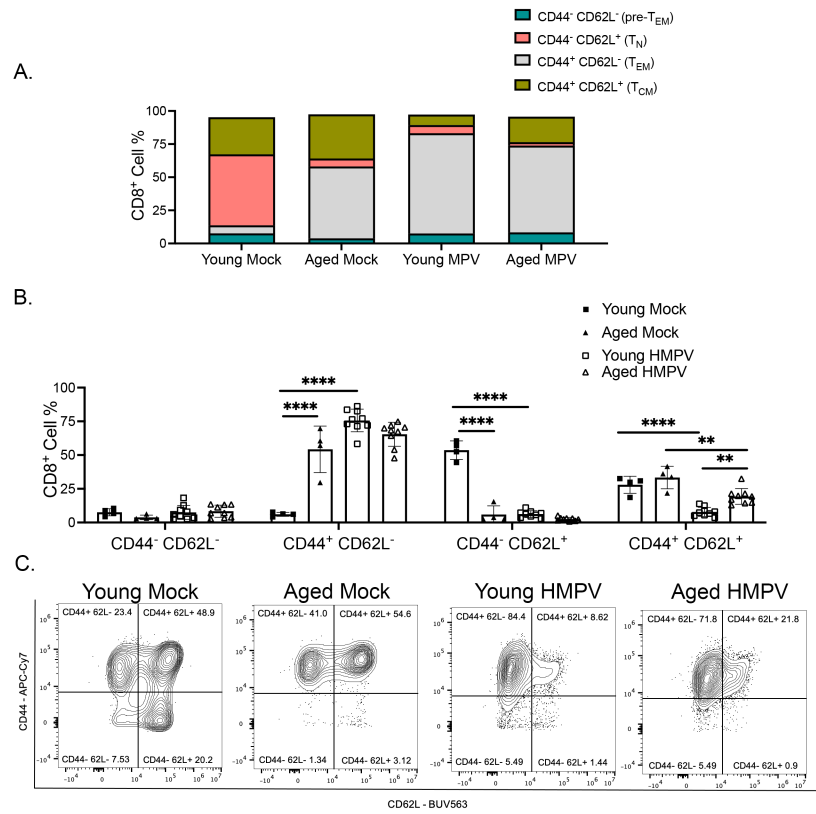

Supplement: Supplementary file 5 — Additional file 5: Supplemental Figure 4. Aged mice had fewer CD44- CD62L+ CD8+ T cells in lung. (A & B) Uninfected aged mice at baseline had significantly fewer naïve CD8+ CD44- CD62L+ T cells (TN) in the lung compared to uninfected young mice. Upon infection, young mice had a robust increase in CD8+ CD44+ CD62L- effector memory cells (TEM) while aged mice had only a modest increase. Bar graphs showing the composition of CD44 and CD62L expression in CD8+ T cells shown in B with raw data points shown in B. (C) Representative flow plots of CD44 and CD62L expression shown for each age group and infection status. [file 12979_2023_365_MOESM5_ESM.pdf]

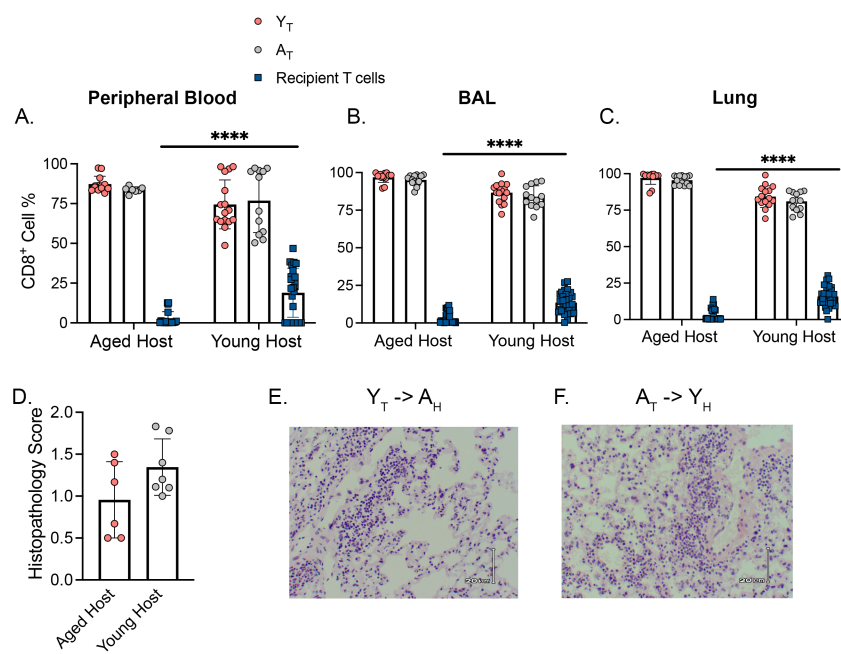

Supplement: Supplementary file 6 — Additional file 6: Supplemental Figure 5. No significant differences in donor T cell engraftment in transplant models. (A-C) Mice were bled by submandibular venipuncture 5 weeks post-irradiation and transplant. Lymphocytes were stained with congenic markers CD45.1 and CD45.2 to determine donor cell engraftment. Graph shows the relative frequencies of donor and recipient CD8+ T lymphocytes from peripheral blood, BAL, and lung, respectively. There was a difference in recipient cells remaining in aged and young hosts, but no signficant differences in donor T cell engraftment. (D) AT -> YH tended to have a higher histopathology score compared to YT -> AH at day 7 p.i. (E & F) Representative histology shown. ****P<0.0001, unpaired t-test. [file 12979_2023_365_MOESM6_ESM.pdf]

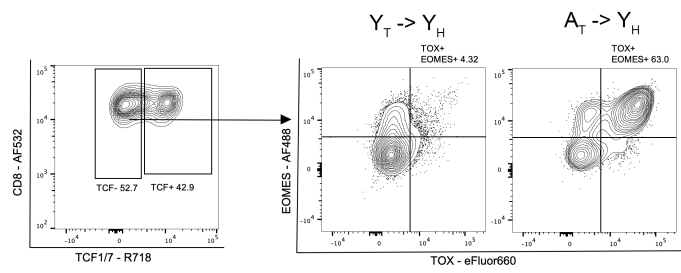

Supplement: Supplementary file 7 — Additional file 7: Supplemental Figure 6. Representative flow plots of TEX CD8+ T cells. [file 12979_2023_365_MOESM7_ESM.pdf]

A.

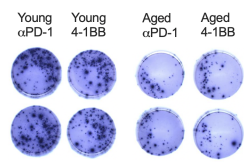

B.

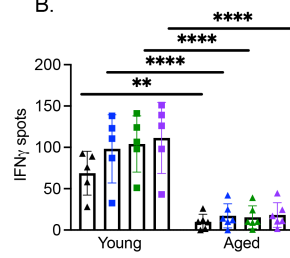

C.

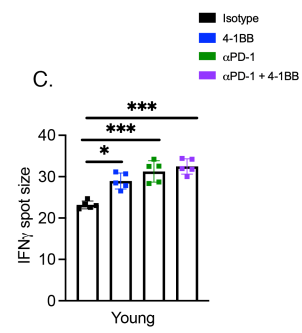

Supplement: Supplementary file 8 — Additional file 8: Supplemental Figure 7. PD-1 blockade, 4-1BB treatment does not improve aged CD8+ T cell function. Ex vivo peptide stimulation of aged or young lung lymphocytes were isolated day 7 p.i. were treated with isotype control antibody, PD-1 blockade, 4-1BB costimulation, or a combination. (A-C) IFNγ spot number was increased and spot size significantly increased in young T lymphocytes treated with 4-1BB, PD-1, and a combination while aged T lymphocytes did not show an improvement in function with any treatment. *P<0.05, ***P<0.001, ****P<0.0001, one-way ANOVA. [file 12979_2023_365_MOESM8_ESM.pdf]
